# Kuru: memories of the NIH years

**DOI:** 10.1098/rstb.2008.4002

**Published:** 2008-11-27

**Authors:** David M. Asher

**Affiliations:** Laboratory of Bacterial, Parasitic and Unconventional Agents, Division of Emerging and Transfusion-Transmitted Diseases, Office of Blood Research and Review, Center for Biologics Evaluation and Research, United States Food and Drug AdministrationCBER FDA HFM-313, 1401 Rockville Pike, Rockville, MD 20852-1448, USA

I was very pleased and honoured when John Collinge and Michael Alpers invited me to share personal recollections about research on kuru and related diseases during the more than 25 years I spent at the National Institutes of Health (NIH). Such reminiscences must be impossibly arbitrary and awkwardly abbreviated when condensed to such a short contribution, so I hope that readers of this paper and others among the many people involved in the work who survive will forgive my inevitable omissions, failures to remember or to credit worthy contributors, and misunderstandings regarding who first conceived of or initiated various aspects of the research. I shall recount my own history at the NIH and summarize a few selected important scientific achievements for which, I believe, NIH research investigators—taken broadly to include some collaborating investigators—were largely responsible, and I shall add a few personal reminiscences.

In July 1966, I arrived at the laboratory of Daniel Carleton Gajdusek in what was then the National Institute of Neurological Diseases and Blindness (NINDB), one of the NIH in Bethesda, Maryland. I came because I was introduced to Carleton's work with kuru by David Lang, infectious disease physician and my mentor during my last year of residency training in paediatrics at the Massachusetts General Hospital in Boston. I came as a Public Health Service (PHS) officer—the Vietnam War having intensified young physicians' interest in PHS-sponsored research—and was later sent, supported by an NIH Special Fellowship sponsored by Carleton, to what was then the Soviet Union. I returned to his laboratory as a civilian research medical officer late in the summer of 1970 and remained with the NIH programme, participating in studies of kuru and related diseases (transmissible spongiform encephalopathies (TSEs) or prion diseases) and other infectious diseases for the next 25 years. At the end of August 1995, I left the NIH to head a small laboratory at the US Food and Drug Administration (FDA) Center for Biologics Evaluation and Research, where I still work today.

By the time I joined Carleton Gajdusek's research group at the NIH, kuru had already been recognized as a novel degenerative brain disease caused by a ‘slow’ scrapie-like infection transmitted to chimpanzees, and kuru's similarities to Creutzfeldt–Jakob disease (CJD) noted. Other animals and cell cultures were already on test as possible assays for infectivity, and collections of tissue samples from cases of CJD had begun. The group's working hypothesis was that the aetiological agent was probably an unconventional virus. Clarence Joseph Gibbs Jr (Joe Gibbs)—working in borrowed space at the US Department of Interior's Patuxent Wildlife Research Center, where the laboratory's primates and other animals were housed—agreed to train me in medical virology and applied primatology; our very close association lasted until Joe's death. I had the bittersweet honour of delivering Joe's final planned public presentation, an invited talk on the pathogenesis of human TSEs before the FDA TSE Advisory Committee, weeks before he died in February 2001. In July 1966, when I arrived at the NIH, Michael Alpers was a Visiting Scientist at NIH and he, Carleton and Joe had just published a monograph on *Slow, latent and temperate virus infections* (Gajdusek *et al.* 1965) that was for many years a definitive source of information on kuru and scrapie and that remains an important historical document. In short, I was neither ‘present at the creation’ of Carleton's NIH laboratory nor did I stay until its end.

Carleton's laboratory at the NIH can be considered to have begun 8 years before I arrived. In 1958 he was appointed Visiting Scientist of NINDB, although, as recorded in correspondence regarding kuru with Joseph E. Smadel (figure 1Farquhar & Gajdusek 1981), NIH had already provided logistical support for fieldwork from the summer of the previous year. Carleton had learned of kuru while a Fellow of the National Foundation for Infantile Paralysis in the Melbourne laboratory of Sir Macfarlane Burnet, who appears to have been considerably less enthusiastic about supporting Carleton's involvement in kuru research than was Smadel. In 1956, Smadel had left the Walter Reed Army Institute of Research, Washington, DC, where he had worked with Carleton from 1952, to become Associate Director of NIH. At the NIH, Smadel was the earliest strong proponent of Carleton's ambitious research plans and occupied a position where he could help realize them; Smadel was clearly responsible for recognizing Carleton's promise, inviting him to the NIH, fostering his early career there, and introducing and recommending him to suitable collaborators at the NIH. Many subsequent NIH administrators were also very supportive, at least until 1994.

Gradually, over the years, the main object of TSE research in the NIH laboratory shifted from kuru to other diseases—probably due to a steady decline in kuru cases after the 1960s and the growing number of other TSE cases referred to the group. But kuru remained the first disease among equals when competing for Carleton's attention. There was no time after which kuru research stopped, and I cannot discriminate meaningfully between those NIH colleagues who worked directly with kuru and those who did not—Carleton tried to engage us all in one aspect of kuru or another.

The NIH laboratory remained under Carleton's direction for approximately 38 years; in January 1995, he announced to NIH administrators his intention to end the research programme (prompting me to take another job later that year), and he left NIH the next year. Staff remaining in the laboratory continued to conduct TSE research for 8 more years, as Joe Gibbs attempted to bring Carleton's programme to an orderly conclusion. With the retirement, in July 2004, of Paul Brown, the last investigator actively engaged in TSE research on the NIH Bethesda campus, Carleton's laboratory ceased to exist, although the NIH continues to maintain some laboratory records and a collection of research samples. During his last years at NIH, Paul Brown became the very effective chairman of the FDA's TSE Advisory Committee, and he remains a sought-after consultant.

## 1. KURU AND CREUTZFELDT-JAkOB DISEASE

### (a) Recognition that kuru was a novel neurodegenerative disease

I am particularly glad to have known Vincent Zigas (see fig. 2 in Gajdusek 2008), who was the first trained physician to recognize kuru as a fatal neurological disease not previously described and to consider the possibility that it might be an infection. But, although Vin might have somewhat resented a relative lack of recognition for those insights, he remained extremely close to Carleton, who appreciated and publicly acknowledged Vin's contribution to the understanding of kuru. Vin, in turn, was grateful to Carleton for overcoming administrative obstacles that had impeded the study of kuru and for planning, organizing and leading as well as tirelessly participating in the research studies at Okapa that led to the seminal descriptions of kuru in the medical literature at the end of 1957 ([Bibr bib23Q4][Bibr bib58Q4]). Vin—who, I believe, spoke four other languages better than his famously mangled English—had a wonderful singing voice and a comic flair; he was a special favourite of our older son, whom he entertained in the mid-1970s with rousing slapstick renditions of the Russian popular song *Kapitan*

### (b) Hypothesis and confirmation that kuru was an infectious disease experimentally transmissible to animals

The hypothesis that kuru, like scrapie, might be transmissible to animals closely related to the natural host but causing overt disease only after exceptionally long incubation periods is generally credited to William Hadlow (1959), who, like Carleton Gajdusek, also went on to study TSEs at the NIH, but more than a thousand miles from Bethesda. At least through last year, Bill Hadlow was still consulting on the pathology of animal TSEs. It is puzzling to me that Bill—having offered the insight that eventually led to solving the mystery of the kuru outbreak—never involved himself in research on kuru or CJD. On the other hand, it seems ironic that the scrapie research unit Bill started at the Rocky Mountain Laboratory of the National Institute of Allergy and Infectious Diseases, in Hamilton, Montana, developed into the only TSE research laboratory remaining in the NIH Intramural Research Program today.

**Figure 2:**
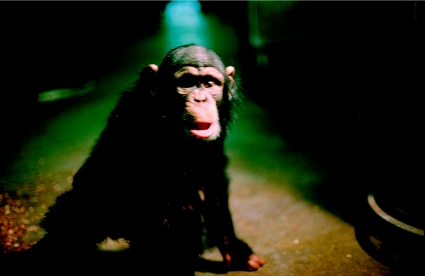
A chimpanzee with early signs of experimental kuru (drooping lower lip) at the Patuxent Wildlife Research Center, 1967 (from the Gibbs archives).

**Figure 4:**
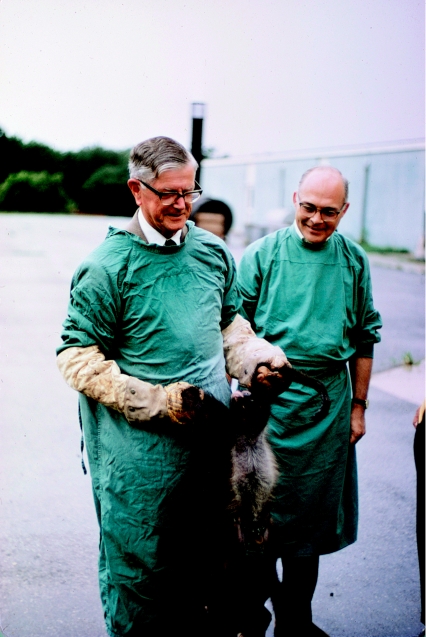
MacFarlane Burnet (left), Joe Gibbs and a spider monkey with experimental kuru, *ca* 1971 (from the Gibbs archives).

The demonstration by Carleton Gajdusek, with Michael Alpers, Joe Gibbs and colleagues, that kuru was experimentally transmissible to chimpanzees (figures 2 and Gajdusek *et al.* 1966), and later to monkeys (figure 4Gajdusek *et al.* 1968), remains, at least in my opinion, the single most important event in uncovering the aetiology and epidemiology of human TSEs. Only someone with both the appetite for high-risk research and unusual persuasive powers of Carleton Gajdusek could have convinced generally conservative NIH administrators to fund (and maintain during several years of negative results) the expensive and exotic programme needed to confirm the kuru–scrapie hypothesis. After 42 years of experience working for the US government, I still cannot imagine how Carleton did it—even with the support of an unusually enthusiastic and powerful champion like Smadel, who died in 1963. Once the programme had begun, the dedicated support of other colleagues, both in the laboratories and in the administrative offices of the NIH, was essential to its later success.

### (c) Hypothesis and confirmation that sporadic Creutzfeldt–Jakob disease (sCJD) was similar in aetiology to kuru

**Figure 5:**
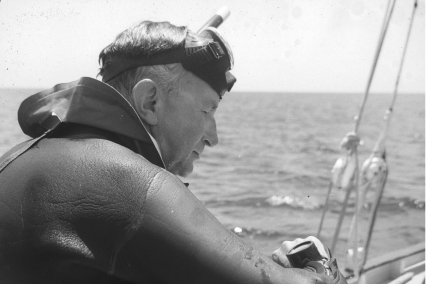
Igor Klatzo in diving gear, preparing to study sharks, in the late 1960s (from the Gibbs archives).

Even before the published descriptions of kuru and Hadlow's observation, and 6 years before the successful transmission of kuru to primates, an NIH investigator took a critical step in understanding the human TSEs. Immediately after examining the histopathology of the first of six brains from people dying with kuru sent to the NIH, Igor Klatzo, chief of neuropathology in NINDB, remarked in a letter to Carleton in Okapa (Farquhar & Gajdusek 1981) that, ‘It [kuru] seems to be definitely a new condition without anything similar described in the literature. The closest condition that I can think of is that described by Jacob [sic] and Creutzfeldt.’ After 2 years, with Gajdusek and Zigas, Klatzo published his observations on the histopathological similarities between kuru and CJD (Klatzo *et al.* 1959) or, as Nevin and others in the UK called it, subacute spongiform encephalopathy (Nevin *et al.* 1960)—the name that Joe Gibbs and Carleton Gajdusek later proposed for the entire class of scrapie-like diseases ([Bibr bib28Q4][Bibr bib27Q4]). Just as I cannot understand why Bill Hadlow never studied kuru, I have wondered why Igor Klatzo, following his first seminal contribution, prepared several more important publications on kuru but then turned his attention to *in vivo* studies of the blood brain barrier in sharks (figure 5) and other models but never returned to the TSEs. Igor spent many years retired not far from Bethesda, and he maintained an indirect connection with TSEs through his friendship with Jurai Cervenak and Larisa Cervenakova, both of whom are colleagues still working with TSEs, Jurai at the FDA and Larisa at the American Red Cross.

**Figure 6:**
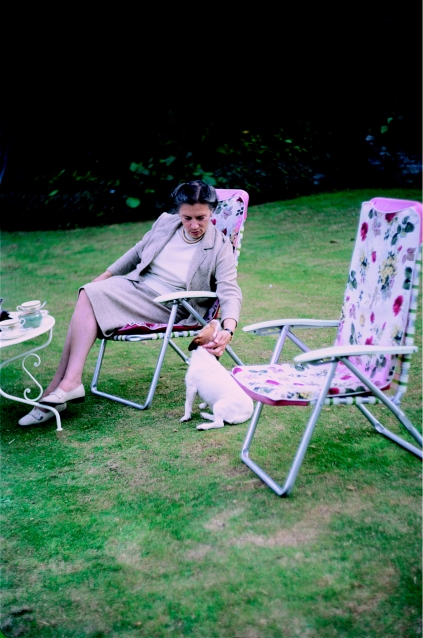
Elisabeth Beck at the Oxford home of H. B. (James) Parry, proponent of the genetic theory for scrapie, 1970 (from the author's collection).

**Figure 7:**
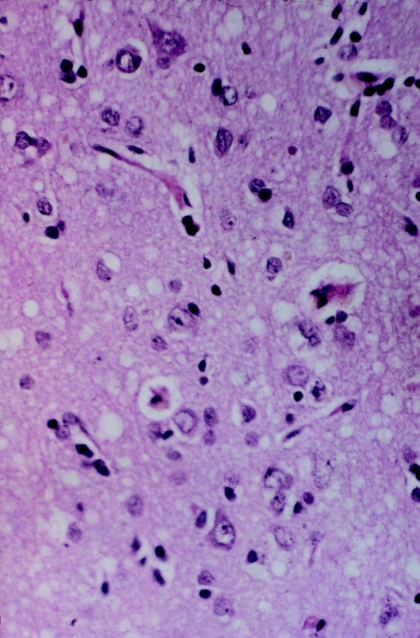
Cortical biopsy of the first patient with CJD sent to the NIH by Elisabeth Beck in 1966 (from Elisabeth Beck's collection).

Igor Klatzo's observation led Elisabeth Beck (figure 6), who studied scrapie (Beck *et al.* 1964) in her laboratory at the Maudsley Institute in London and later compared the neuropathology of kuru in humans with the experimental disease in chimpanzees and monkeys (Beck *et al.*^1966^,^1969^), to obtain and send the first samples of brain tissues from patients with CJD (figure 7) to the NIH laboratory. CJD was successfully transmitted from those samples, and later many others, to chimpanzees (Gibbs *et al.* 1968), monkeys, cats and rodents, demonstrating that TSEs affected people throughout the world, not just in Papua New Guinea, and that the spectrum of scrapie-like infections in humans must be greater than first suspected. Until late in her life, Elisabeth continued to study the histopathology and eventually the ultrastructural pathology of human TSEs (Beck *et al.* 1975), as she had studied scrapie earlier. Elisabeth doted on Carleton and Joe, but not enough to overcome her intolerance of jet lag and especially her great distaste for the hot and humid weather that plagues Washington from late spring through early autumn; during her last summer visit, she announced that if we ever wanted to see her again it had to be in London or at her home in Epsom, but not in Washington. Her aversion to travel apparently extended only westward; she loved walking in the mountains near Arosa, Switzerland each summer. After retiring from the Maudsley, Elisabeth enjoyed a year working in Germany, only one time zone away from Epsom, gratefully accepting an honorary medical degree and trying to forget the humiliation of her exile during World War II.

It was unquestionably the demonstration by the NIH group that CJD was a second human TSE, so that kuru did not just represent a one-off exotic disappearing disease resulting from cannibalism—and that there existed a whole class of previously unsuspected infectious agents capable of transmitting non-inflammatory progressive neurological diseases years after exposure—that was the discovery that resulted in the Nobel Prize in Physiology or Medicine being awarded to Carleton in 1976 (Gajdusek 1977).

### (d) Demonstration that familial CJD and Gerstmann–Sträussler–Scheinker syndrome were associated with aetiological agents similar to that causing sporadic CJD

Walter R. Kirschbaum, like Elisabeth Beck, a product of the classical German school of neurology and fortunate to have left Germany before the War (in his case emigrating to Chicago), first described CJD in members of the famous Backer family (Kirschbaum 1924)—a huge German kindred with familial CJD expressed in an autosomal dominant pattern (Meggendorfer 1930). Kirschbaum, author of a prodigious summary of the first 150 published cases of ‘Jakob–Creutzfeldt disease’ (Kirschbaum 1968), preferred the names in reverse chronological order (perhaps doubting that Creutzfeldt's case was really the same disease). He was sufficiently impressed by the model of kuru to comment presciently that ‘The slow-virus concept applicable to other neurological diseases should not be dismissed from pathogenetic considerations of J–C disease.’ Prof. Kirschbaum visited the NIH laboratories in the early 1970s. He was clearly a fine neurologist but exceptionally sensitive to imagined slights; I recall his waiting in a line for taxis to a dinner and rebuking me that ‘This lady you seated first but me you leave!’ I had to explain patiently that the lady was my wife.

It was Colin Masters during his years at the NIH who first noted that some cases of CJD that had transmitted disease to animals were familial (Masters *et al.* 1979) and that some of the familial transmitted cases were better diagnosed as examples of the Gerstmann–Sträussler–Scheinker (GSS) syndrome (Masters *et al.* 1981). I am still not sure that the somewhat arbitrary distinction between familial CJD and GSS is important for understanding the aetiology of TSE infections and effects of host genetics, but that is a small point. I consider the demonstration that familial as well as sporadic human TSEs are associated with, if not caused by, infectious agents similar to those responsible for sporadic CJD to be a major accomplishment of the NIH TSE programme that began with kuru.

### (e) Use of animal assays to elucidate the tissue distribution of the infectious agent in kuru and TSEs

The elaborate efforts of Carleton Gajdusek, Joe Gibbs and colleagues to address the hypothesis that other chronic progressive neurological diseases might be caused by agents similar to those transmitting kuru and CJD/GSS uncovered no unsuspected TSEs among the more common ailments, such as Alzheimer's disease or multiple sclerosis. However, the studies—sometimes ridiculed then as ‘Noah's ark’ transmission attempts—resulted in another contribution: they helped to reveal the distribution of infectivity in various tissues of people dying with kuru or CJD, information that remains important today (Brown *et al.* 1994). The NIH studies were the first to demonstrate that infectivity in the blood of an animal model was highly associated with nucleated cells (Kuroda *et al.* 1983), and two alumni of the NIH group, Paul Brown and Robert Rohwer, and colleagues, later extended that work to show that plasma, but probably not erythrocytes or platelets, also contained a large fraction of the total infectivity in blood ([Bibr bib16Q4][Bibr bib31Q4]). Bob Rohwer, now Professor at the University of Maryland/Baltimore Veterans Administration Health Center and head of the Baltimore Research and Education Foundation, has since gone on to develop an interesting candidate device for removing infectivity from blood (Gregori *et al.* 2006**).

## 2. Recognition of iatrogenic transmission of CJD

Another contribution resulted from a collaborative effort between Christoph Bernoulli, then at the Zurich Kantonsspital, and the NIH kuru research group. Chris recognized that a cortical electrode probe had transmitted CJD from an older patient to two young people undergoing epilepsy surgery (Bernoulli *et al.* 1977). An earlier clinical report described a transmission by a transplanted cornea (Duffy *et al.* 1974), but iatrogenic CJD from contaminated instruments—though suggested indirectly by Nevin's case series (Nevin *et al.* 1960)—had not been reported. Chris enlisted the assistance of our group at the NIH, both to prepare his report for publication and to assay the actual implicated probe for residual infectivity. The dramatic transmission of CJD by implanting pieces of the probe in the frontal lobe of a chimpanzee (Gibbs *et al.* 1994) eliminated any doubt that conventional cleaning and sterilization had failed to remove residual infectivity. Similar studies assaying retention lots of human cadaveric pituitary growth hormone (Gibbs *et al.* 1993) served a similar purpose in confirming experimentally the clinical evidence of iatrogenic transmissions of CJD by that product. I hope that Chris Bernoulli finally made his peace with what happened in Zurich so many years ago and takes comfort in having played a critical role in promoting awareness of the potential risk from contamination of neurosurgical instruments with TSE agents. It is probably not coincidental that, in spite of increased surveillance, no case of iatrogenic CJD has been attributed to contaminated surgical instruments for many years (Foncin *et al.* 1980).

## 3. Demonstration of the unusual physical properties of the TSE agents

The NIH kuru research programme also contributed to the improved understanding of the unique physical properties of the TSE agents. The hypothesis that scrapie was caused by a unique pathogen with physical properties so unusual as to be inconsistent with a nucleic acid encoding the synthesis of its components and its self-replication was already circulating when I arrived in Carleton's group in 1966. Pattison and Sansom had concluded, in 1964, that the scrapie agent was dialysable through pores too small to admit the passage of viruses ([Bibr bib50Q4][Bibr bib48Q4][Bibr bib49Q4]) and Alper and colleagues had just reported the results of her first studies with ultraviolet light inactivation that they interpreted as inconsistent with a nucleic acid component of the agent (Alper *et al.* 1966). Griffith offered a number of possible mechanisms by which a nucleic-acid-free agent might encode pathogenic self-replicating information (Griffith 1967). NIH investigators were involved in confirming and extending those observations to other TSE agents and ionizing radiation ([Bibr bib42Q4][Bibr bib41Q4]), while warning that estimates of maximal size of nucleic acid in TSE agents based on inactivation by irradiation were probably too small (Rohwer & Gajdusek 1980; Rohwer 1984**,^1986^). Bob Rohwer's studies at the NIH suggested that most infectivity of the scrapie agent was, contrary to previous reports, destroyed by boiling (Rohwer 1984**) and that its resistance to heat inactivation resulted from a very small resistant fraction; my own modest contribution was to show that drying on to surfaces seemed to protect infectivity from inactivation (Asher *et al.*1986**,^1987^), a finding of practical importance (Grobben *et al.* 2005) that I underestimated at the time. The discovery of the prion protein, the elucidation of its role in susceptibility to TSE infection and the genetic basis of familial TSEs, followed by the full elaboration of what came to be known as the ‘prion’ or ‘all-protein’ hypothesis for the probable nature of the transmissible agent, all issued from the Nobel prize-winning work of Stanley Prusiner and colleagues (Bolton *et al.* 1982; Prusiner 1982,1997), although we later recognized the use of the newly described abnormal prion protein for the improved diagnosis of TSEs (Brown *et al.* 1986). NIH staff were also involved in the earliest descriptions of unusual tubulovesicular particles commonly associated with TSEs (David-Ferreira *et al.* 1968) that sceptics of the prion hypothesis still propose as possible nucleic-acid-containing aetiological agents of TSEs (Manuelidis *et al.* 2007). Until the very end of the NIH laboratory, we never reached consensus in our views about the all-protein hypothesis for the probable nature of TSE agents.

## 4. Collaborative nature of research on kuru and other TSEs at the NIH

During those years, in addition to resident staff and many predoctoral and postdoctoral fellows, a series of visiting scientists joined the kuru research programme for periods of months or years. I can remember and name at least 100 of the colleagues whom I worked with or who were part of Carleton's team in the laboratory during my time at the NIH. A dozen or more of them have died, some have retired but most are still professionally active and several are internationally prominent scientists. Of the many support and technical staff involved in kuru research at the NIH, I particularly remember Mint Basnight, Alfred Bacote, Don Cameron Jr, Olive Childers, Laura Kreiss, Steven Ono, Kitty Pomeroy, Linda Poole, Ginny Rousculp, John and Dory Runman, Gary Stone, Michael Sulima and LaDonna Tavel; Ivan Mbaginta'o, Marion Poms (who was Carleton's devoted secretary from his arrival at the NIH in 1958), Nancy Rogers and Lucille Gilbert have died. Then there are the enormous number of collaborating scientists within the NIH and elsewhere who did not work in the kuru laboratory but contributed in other important ways, supporting Carleton and Joe and maintaining the intellectual ferment that enabled success. It would be a daunting task to try and acknowledge everyone who contributed to this remarkable enterprise.

## 5. Continuing influence of the NIH kuru research in my own life

Since 1996, Pedro Piccardo, Kitty Pomeroy (both NIH alumni), others at the FDA and I, with Larisa Cervenakova, also an alumna of the NIH TSE programme and now directing her own laboratory at the American Red Cross, have continued to study those same diseases and some of the same issues that were the object of my earliest efforts when I came to work with Joe Gibbs, Michael Alpers and Carleton Gajdusek at the NIH 42 years ago. Our group at the FDA has been evaluating the resistance of cell cultures ([Bibr bib4Q4][Bibr bib57Q4]), in particular cells used as ‘substrates’ for generating vaccines and other biological products, to infection with the agent of bovine spongiform encephalopathy and other TSE agents. I am also slowly completing applied work begun 28 years ago on methods for decontaminating TSE agents dried onto surfaces (Asher *et al.*1986**,^1987^). I hope that my younger colleagues will soon expand our studies in the FDA to evaluate candidate ante-mortem diagnostic tests for TSEs and practical devices for removing the infectious agents from blood and other fluids used in the manufacture of biologicals. We also serve FDA as sources of historical and technical information about TSE infections (Buckner & Asher 2008) and their implications for regulatory actions and public health policies affecting the safety of blood components, plasma derivatives, vaccines, medical devices and other medical products (Asher 1999**). So it is clear that early experience with kuru research at the NIH influenced the rest of my professional life. It indirectly benefited my personal life as well, but that story I leave for another time and place.

## Figures and Tables

**Figure 1:**
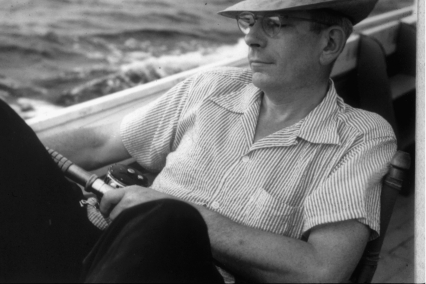
Joseph E. Smadel, sometime in the 1950s (from the archives of Clarence J. Gibbs, Jr and the Laboratory of Central Nervous System Studies, NIH).

**Figure 3:**
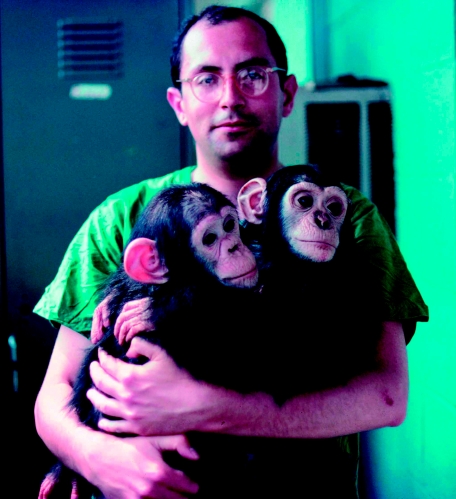
The author and friends at the Patuxent Wildlife Research Center, *ca* 1968 (from the Gibbs archives).
